# Major Role of S-Glycoprotein in Providing Immunogenicity and Protective Immunity in mRNA Lipid Nanoparticle Vaccines Based on SARS-CoV-2 Structural Proteins

**DOI:** 10.3390/vaccines12040379

**Published:** 2024-04-02

**Authors:** Evgeniia N. Bykonia, Denis A. Kleymenov, Vladimir A. Gushchin, Andrei E. Siniavin, Elena P. Mazunina, Sofia R. Kozlova, Anastasia N. Zolotar, Evgeny V. Usachev, Nadezhda A. Kuznetsova, Elena V. Shidlovskaya, Andrei A. Pochtovyi, Daria D. Kustova, Igor A. Ivanov, Sergey E. Dmitriev, Roman A. Ivanov, Denis Y. Logunov, Alexander L. Gintsburg

**Affiliations:** 1 Federal State Budget Institution “National Research Centre for Epidemiology and Microbiology Named after Honorary Academician N. F. Gamaleya” of the Ministry of Health of the Russian Federation, Moscow 123098, Russia; mne10000let@yandex.ru (D.A.K.); andreysi93@ya.ru (A.E.S.); lenok27microb@gmail.com (E.P.M.); sofya_dadashyan@mail.ru (S.R.K.); zolotar.ananas@gmail.com (A.N.Z.); evgenyvusachev@gmail.com (E.V.U.); nadyakuznetsova0@yandex.ru (N.A.K.); lenitsa@gmail.com (E.V.S.); a.pochtovyy@gmail.com (A.A.P.); kustovad70@gmail.com (D.D.K.); chai.mail0@gmail.com (I.A.I.); sergey.dmitriev@belozersky.msu.ru (S.E.D.); ldenisy@gmail.com (D.Y.L.); gintsburg@gamaleya.org (A.L.G.); 2 Department of Virology, Lomonosov Moscow State University, Moscow 119234, Russia; 3 Department of Medical Genetics, Federal State Autonomous Educational Institution of Higher Education I M Sechenov First Moscow State Medical University of the Ministry of Health of the Russian Federation (Sechenov University), Moscow 119991, Russia; 4 Shemyakin-Ovchinnikov Institute of Bioorganic Chemistry of the Russian Academy of Sciences, Moscow 117997, Russia; 5 Belozersky Institute of Physico-Chemical Biology, Lomonosov Moscow State University, Moscow 119234, Russia; 6 Faculty of Bioengineering and Bioinformatics, Lomonosov Moscow State University, Moscow 119991, Russia; 7 Translational Medicine Research Center, Sirius University of Science and Technology, Sochi 354340, Russia; ivanov.ra@talantiuspeh.ru; 8 Infectiology Department, I. M. Sechenov First Moscow State Medical University, Moscow 119991, Russia

**Keywords:** SARS-CoV-2, mRNA vaccine, lipid nanoparticles, immunogenicity, efficacy

## Abstract

SARS-CoV-2 variants have evolved over time in recent years, demonstrating immune evasion of vaccine-induced neutralizing antibodies directed against the original S protein. Updated S-targeted vaccines provide a high level of protection against circulating variants of SARS-CoV-2, but this protection declines over time due to ongoing virus evolution. To achieve a broader protection, novel vaccine candidates involving additional antigens with low mutation rates are currently needed. Based on our recently studied mRNA lipid nanoparticle (mRNA-LNP) platform, we have generated mRNA-LNP encoding SARS-CoV-2 structural proteins M, N, S from different virus variants and studied their immunogenicity separately or in combination in vivo. As a result, all mRNA-LNP vaccine compositions encoding the S and N proteins induced excellent titers of RBD- and N-specific binding antibodies. The T cell responses were mainly specific CD4^+^ T cell lymphocytes producing IL-2 and TNF-alpha. mRNA-LNP encoding the M protein did not show a high immunogenicity. High neutralizing activity was detected in the sera of mice vaccinated with mRNA-LNP encoding S protein (alone or in combinations) against closely related strains, but was undetectable or significantly lower against an evolutionarily distant variant. Our data showed that the addition of mRNAs encoding S and M antigens to mRNA-N in the vaccine composition enhanced the immunogenicity of mRNA-N and induced a more robust immune response to the N protein. Based on our results, we suggested that the S protein plays a key role in enhancing the immune response to the N protein when they are both encoded in the mRNA-LNP vaccine.

## 1. Introduction

Since the beginning of the COVID-19 pandemic, there have been several global waves of increased incidence caused by the rapidly evolving SARS-CoV-2 variants: Alpha (B.1.1.7), Beta (B.1.351), Gamma (P.1), Delta (B.1.617.2), Omicron (B.1.1.529), and others. Mutations in the gene encoding the SARS-CoV-2 spike protein have been found to contribute to the immune evasion and/or increased transmissibility of the virus, resulting in the reduced effectiveness of existing approved vaccines that primarily target the viral S protein to produce a potent neutralizing antibody response [1,2,3].

The WHO Technical Advisory Group on COVID-19 Vaccine Composition in the WHO Global COVID-19 Vaccination Strategy, published in June 2022, called for future boosters to be compatible with circulating SARS-CoV-2 variants and/or have a broad immunogenicity against new variants of concern [4]. As a result, in the fall of 2022 Pfizer/BioNTech and Moderna produced booster bivalent mRNA vaccines encoding the spike proteins of the wild-type SARS-CoV-2 variant and one of the Omicron variants BA.1 or BA.4/5 [5,6]. These updates to the vaccine antigen composition led to enhanced vaccine-induced immune responses to circulating SARS-CoV-2 variants [7].

However, soon after Omicron sublineage XBB was first identified in August 2022, the XBB.1 descendants (i.e., XBB.1.5, XBB.1.16, XBB.1.9) became dominant globally in the first half of 2023 [8,9,10].

As a result, sera from individuals who have received two, three, or four doses of index virus-based vaccines or a booster dose of a bivalent mRNA vaccine (containing BA.1 or BA.4/5) show substantially lower neutralizing antibody titers against XBB.1 descendent lineages, as compared to titers specific to the antigens included in the vaccine [8]. Consequently, new updated COVID-19 vaccines developed by Pfizer-BioNTech and Moderna were approved by the Food and Drug Administration (FDA) in mid-September 2023 and are recommended by the Centers for Disease Control and Prevention (CDC). These updated vaccines consist of a monovalent composition, with mRNA coding for the S protein of Omicron XBB.1.5 only [11].

However, there is ongoing and considerable genetic and antigenic evolution of SARS-CoV-2, posing new challenges for the global community. To date, new subvariants of the virus have already predominated, and highly mutated ones (i.e., BA.2.86) have also emerged [12,13]. It is too early to say whether the newly adapted boosters will protect against these variants. Preliminary data suggest the potential utility of the monovalent XBB.1.5 mRNA boosters, but this is an area of ongoing research [12].

Thus, continuous vaccine updates provide a high level of protection against severe disease and mortality caused by all circulating variants of SARS-CoV-2, but it declines over time due to the ongoing evolution of the virus. One possible approach to improving the durability and breadth of protection is to include more conserved antigens in the vaccine in addition to the S protein.

Natural infections are known to provide superior immunity and broad protection compared to vaccines [14]. T cell responses to SARS-CoV-2 indicate that there are many potential CD4^+^ and CD8^+^ T cells specific for both structural and non-structural proteins of the virus. In particular, the M, S, and N proteins are codominant in natural infection, each being recognized by CD4^+^ and CD8^+^ T cells in the vast majority of human COVID-19 survivors [15,16,17]. T cell responses develop early and play a central role in the control of SARS-CoV-2, correlating with protection [18]. Thus, the inclusion of more conserved antigens (e.g., N and M proteins) in the vaccine may provide broader protection and long-lasting immunity [19,20,21].

We have recently investigated an mRNA lipid nanoparticle (mRNA-LNP) platform and found that it enables the efficient long-term expression of an encoded gene in vivo after both intramuscular and intravenous application. The platform has been extensively characterized using firefly luciferase (Fluc) as a reporter [22]. Based on this platform, in this study we generated mRNA-LNP-vaccine candidates that encoded structural proteins (M, N, and S proteins) from different SARS-CoV-2 variants. We used the gene sequences of two early variants (Wuhan and Delta) and two later ones (Omicron BA.5.3.1 and XBB.1). The aim of this study was to evaluate the immunogenicity and protective efficacy of each antigen encoded in an mRNA vaccine alone or in combination with each other and to make possible recommendations regarding vaccine composition against SARS-CoV-2 variants of concern (VOCs).

## 2. Materials and Methods

### 2.1. mRNA Synthesis and mRNA-LNP Formulation

mRNAs encoding following antigens were synthesized:

- S protein of wild-type strain SARS-CoV-2 (Wuhan);
- S protein of Delta variant (B.1.617.2);
- S, N and M proteins of Omicron variant (BA.5.3.1);
- S protein of Omicron variant (XBB.1).

mRNA-LNP preparations were produced as previously described [22]. Briefly, the coding regions of the investigated antigens (M, N, S proteins) were cloned into a vector for in vitro transcription (IVT) based on the pJAZZ-OK linear bacterial plasmid. Cloning and plasmid production were performed using *E. coli* BigEasy™-TSA™ Electrocompetent Cells (Lucigen, Middleton, WI, USA). All cloning procedures were verified by Sanger sequencing using BigDye^®^ Terminator v3.1 Cycle Sequencing kit on 3500 Genetic Analyzer (Applied Biosystems, Foster City, CA, USA). The pDNA for IVT were isolated, purified from a culture of the *E. coli* using a Plasmid Maxi-Kit (Qiagen, Hilden, Germany) and then digested with BsmBI-v2.

IVT was performed as described earlier [22]: the 100 μL reaction volume contained 3 μg of DNA template, 3 μL T7 RNA polymerase (Biolabmix, Novosibirsk, Russia) and 10×Buffer (TriLink BioTechnologies, San Diego, CA, USA), 4 mM trinucleotide cap 1 analog ((3′-OMe-m7G)-5′-ppp-5′-(2′-OMeA)pG) (Biolabmix, Novosibirsk, Russia), 5 mM m^1^ΨTP (Biolabmix, Russia) replacing UTP, and 5 mM GTP, ATP, CTP. After 2 h incubation at 37 °C, 6 μL DNase I (Thermo Fisher Scientific Inc., Waltham, MA, USA) was added for an additional 15 min, followed by a mRNA precipitation with 2M LiCl (incubation for 1 h on ice and centrifugation for 10 min at 14,000× *g*, 4 °C) and carefully washed with 80% ethanol. RNA integrity was assessed by electrophoresis in 8% denaturing PAGE.

In vitro transcribed mRNAs were encapsulated in LNPs as described earlier with some modifications [22]. Briefly, lipids were dissolved in ethanol at molar ratios of 46.3:9:42.7:1.6 (ionizable lipid (ALC-0315)–distearoyl PC–cholesterol–PEG-lipid (ALC-0159)). The lipid mixture was combined with 10 mM sodium citrate buffer (pH 3.0) containing mRNA (0.2 mg/mL) at a volume ratio of 3:1 (aqueous phase:organic phase) using a microfluidic cartridge technology in NanoAssemblr Ignite device (Precision NanoSystems, Vancouver, BC, Canada). Formulations were then dialyzed against PBS buffer (VWR Life Science, Radnor, PA, USA) through a 3.5 K MWCO Slide-A-Lyzer dialysis cassette (Thermo Fisher Scientific Inc., Waltham, MA, USA), followed by being passed through a 0.2 μm filter and stored at 4 °C until use.

The diameter and size distribution of the mRNA-LNP was measured by dynamic light scattering (DLS) using a Zetasizer Nano ZS instrument (Malvern Panalytical, Worcestershire, UK). The mRNA-LNP suspension was diluted in water 20 times and loaded into a cuvette for measurement at 25 °C. LNPs in PBS were read using the solvent parameter ‘water’. One measurement consists of three readings and each reading is derived from 13 acquisitions. The DLS data presented for each mRNA-LNP preparation are the average value of these three readings.

The mRNA encapsulation efficiency and concentration were determined by SYBR Green dye (SYBR Green for PCR, Lumiprobe, Moscow, Russia) followed by fluorescence measurement. Briefly, mRNA-LNP samples were diluted with TE buffer (pH 8.0) in the absence or presence of 2% Triton-X-100 in a black 96-well plate. Standard mRNA was serially diluted with TE buffer in the absence or presence of 2% Triton-X-100 to generate standard curves. Then, the plate was incubated for 10 min followed by the addition of SYBR Green dye (100 times diluted) to each well to bind RNA. Fluorescence was measured at 454 nm excitation and 524 nm emission using Varioscan LUX (Thermo Fisher Scientific Inc., Waltham, MA, USA). The concentrations of mRNA after LNP disruption by Triton-X-100 (C _total mRNA_) and before LNP disruption (C _outside mRNA_) were determined using corresponding standard curves. The concentration of mRNA loaded into the LNP was determined as the difference between the two concentrations multiplied by the dilution factor of the original sample. Encapsulation efficiency was calculated using Formula (1):

(1) $$Encapsulation\;efficiency\;(\%)\;=\frac{C(total\;mRNA)-C(outside\;mRNA)}{C(total\;mRNA)}\times 100$$

### 2.2. Cell Transfection

The expression of each vaccine-encoded viral protein from the in vitro transcribed mRNA was confirmed in HEK293T cells (ATCC). Cell transfection was performed as previously described [22]. Briefly, 3 × 10^4^ cells per well were transferred into the 96-well plates (Greiner BioOne, Longwood, FL, USA) in 75 μL of DMEM (Paneco, Moscow, Russia) supplemented with 10% FBS (HyClone, San Angeo, TX, USA) in the presence of 50 U/mL penicillin and 50 μg/mL streptomycin (both from Paneco, Moscow, Russia). The cell culture was incubated at 37 °C in 5% CO_2_ atmosphere. The next day, the cell culture was transfected with the mRNA. For this, 30 ng of mRNA in 20 μL Opti-MEM (Gibco, Thermo Fisher Scientific Inc., Waltham, MA, USA) per well was mixed with a solution of 0.06 μL of GenJector-U (Molecta, Moscow, Russia) in 3 μL Opti-MEM (per well), incubated for 15 min, and added to the cells. Transfected cells were incubated at 37 °C with 5% CO_2_ for 24 h. Then, cells were collected, centrifuged at 300× *g*, for 5 min, and at 4 °C. Supernatant was removed following the addition of 100 µL of NENT lysis buffer to the cell pellet and incubation on ice for 30 min. The cell lysate was centrifuged at 13,000× *g,* for 5 min, at 4 °C, and the supernatants were subsequently analyzed.

### 2.3. Detection of Protein Production

Protein production was confirmed by in-house bead-based immunoassay (Luminex Corp., Austin, TX, USA). MagPlexTM-C carboxylated microspheres (beads) were used for conjugation. Mouse monoclonal N- and S-specific IgG antibodies (clone RBD5308 and clone C518, Hytest, Moscow, Russia) and rabbit polyclonal M-specific IgG (AtaGenix Laboratories, Wuhan, China) were conjugated to beads (each to their own beads’ region) according to the protocol for two-step carbodiimide reaction in the Luminex Cookbook [23]. Briefly, the coupling procedure was performed as follows: 1 × 10^6^ beads from one region were activated with 10 µL of 50 mg/mL EDC and 10 µL of 50 mg/mL s-NHS (both from Thermo Fisher Scientific Inc., Waltham, MA, USA) in 80 µL activation buffer (0.1 M NaH_2_PO_4_, pH 6.2) for 20 min at 25 °C. After that, the activated beads were washed twice and resuspended in 500 µL of coupling buffer (50 mM MES, pH 5.0) with the 10 µg of specific antibodies. The incubation was conducted for 2 h at RT in the dark and mixed on a Rotamix RM-1L rotator (ELMI, Riga, Latvia). After the coupling procedure, the beads were washed three times, resuspended in 1 mL PBS-TBN buffer (PBS, 0.1% BSA, 0.02% Tween-20, 0.05% NaN_3_, pH 7.4), quantified using an automatic cell counter TC-20 (Bio-RAD, Hercules, CA, USA), and stored in the dark at 2–8 °C until use.

The immunoassay was performed in monoplex as previously described with some modifications [24]. For reference, standard recombinant proteins (N, M, or RBD) were used (see below). The reference standard was 3-fold serially diluted over 8 points using PBS-TBN buffer to establish the calibration curve. Standard dilutions and cell lysate were 20-fold diluted with PVXC buffer (PBS, 0.8% PVP, 0.1% casein, 0.5% PVA, 0.05% NaN_3_). Then, 2500 microspheres in 80 µL PBS-TBN buffer per well were incubated with 20 µL of sample (final dilution of 1:100 for all samples in the well) for 60 min at 37 °C in the dark on a rotating shaker (800 rpm). Then, beads were washed twice with 200 µL PBS-TBN buffer using an Agilent BioTek 405 TS Microplate Washer magnetic plate separator (Agilent Technologies, Santa Clara, CA, USA). Next, 100 µL of biotinylated secondary antibodies to corresponding proteins in PBS-TBN buffer (4 µg/mL) was added to microspheres and incubated for 60 min in the same conditions. The following secondary antibodies were used: monoclonal N- and S- specific IgG antibodies (rabbit IgG, clone C706 and mouse IgG, clone RBD5305, respectively, Hytest, Russia), and rabbit polyclonal anti-M-protein antibodies (#PODTC5, AtaGenix Laboratories, Wuhan, China). Then, the beads were washed twice in the same manner. Next, 100 µL of SAPE (Thermo Fisher Scientific Inc., Waltham, MA, USA) in PBS-TBN buffer (4 µg/mL) was added to the microspheres and incubated for 30 min in the same conditions. After a final wash step, the beads were resuspended in 100 µL of PBS-TBN and analyzed on a MAGPIX instrument. For each identified analyte, the MFI value was converted to ng/mL by interpolation from a 5-parameter logistic (5-PL) curve of the reference standard using the MILLIPLEX^®^ Analyst 5.1 software (The Life Science business of Merck KGaA, Darmstadt, Germany).

### 2.4. Mouse Immunization and SARS-CoV-2 Challenge

All animal experiments were approved by the Institutional Animal Care and Use Committee (IACUC) of the Federal Research Centre of Epidemiology and Microbiology named after Honorary Academician N.F. Gamaleya and were performed under Protocol #56 from 31 July 2023.

Vaccine immunogenicity was evaluated in 4–5-week-old females BALB/c mice (The Federal Medical-Biological Agency or FMBA, Stolbovaya breeding nursery, Russia). For immunogenicity, nine groups of mice (six per group) were immunized intramuscularly at week 0 (prime, V1) and week 3 (boost, V2), respectively. Mice groups were immunized with either PBS (control group), mRNA-S-Wuhan (5 μg), mRNA-S-Delta (5 μg), mRNA-S-Omicron (5 μg), mRNA-N (5 μg), mRNA-M (5 μg), or a combination of these mRNAs (5 μg for each mRNA component). The vaccine or PBS was administered at 100 μL per injection. Blood and serum samples were collected 2 weeks after first dose (V1) and 2 weeks after second vaccination (V2) to measure vaccine-induced binding antibody response. All mice were euthanized 2 months after the booster (V2). Blood and serum and spleen samples were collected for analyses of vaccine-induced humoral (neutralizing antibodies) and cellular immune responses.

Vaccine efficacy was studied in a SARS-CoV-2 challenge on 6-week-old transgenic B6.Cg-Tg(K18-ACE2) mice (“Medgamal” subsidiary of N. F. Gamaleya Federal Research Center for Epidemiology and Microbiology). Mice were divided into five groups of 16 individuals per group (8 males and 8 females). Mice groups were immunized with either PBS (control group), mRNA-S XBB.1 (5 μg), or combined mRNA vaccines (5 μg for each mRNA component) − mRNA-S XBB.1 + mRNA-N, or mRNA-S-Wuhan + mRNA-S-XBB.1, at week 0 (prime) and week 3 (boost), respectively. The vaccine or PBS was administered at 100 μL per injection. Two weeks after the booster dose, all mice were transferred to a animal biosafety level 3 facility (ABSL-3). Then, after two weeks, mice were challenged intranasally with an 10^5^ TCID_50_ SARS-CoV-2 BF.7 strain (GISAID EPI_ISL_16348906). Four days after the viral challenge, six mice from each group were euthanized and equivalent portions of the lung tissues were collected for quantification of SARS-CoV-2 viral loads.

### 2.5. Binding IgG Detection by ELISA

Vaccine-induced binding IgG antibodies to the N and M proteins and the receptor-binding domain (RBD) of the S protein were measured by “in-house” enzyme-linked immunosorbent assay (ELISA) as previously described [25]. Briefly, plates (Xema, Moscow, Russia) were coated with recombinant RBD of different SARS-CoV-2 variants (0.5 μg/mL; EVV00312 (Delta) and EVV00329 (BA.5), (AntibodySystem, Schiltigheim, France); 8COV1 (Wuhan), HyTest, Moscow, Russia), N protein (1 μg/mL “In-house” production), or M protein (1 μg/mL; ATEP02465COV, AtaGenix Laboratories, Wuhan, China). The next day, the plates were blocked for 2 h at room temperature with an S002X buffer (Xema, Moscow, Russia). After the blocking buffer was removed, serially diluted serum samples were added into the wells and incubated for 1 h at 37 °C (initial dilution, 1:100; 1:2 serial dilution in ELISA buffer S011 (Xema, Moscow, Russia). Plates were washed 3 times and incubated with 100 μL HRP-conjugated anti-mouse IgG secondary antibody (L20/01; HyTest, Moscow, Russia) for 1 h at 37 °C. After a final wash, plates were visualized using a chromogen–substrate solution R055 (Xema, Moscow, Russia). After 10 min, the reaction was stopped using 10% HCl. Plates were read at 450 nm wavelength within 15 min by using a Multiscan reader (Thermo Fisher Scientific Inc., Waltham, MA, USA). Binding IgG endpoint titers (EPTs) for each sample were calculated.

### 2.6. ICS and Flow Cytometry

Isolated mouse splenocytes were washed with fluorescence-activated cell sorting (FACS) buffer (PBS, 0.5% BSA) and resuspended in RPMI-1640 supplemented with 10% FBS, 2-mercaptoethanol, penicillin–streptomycin, and L-glutamine. Then, 2 × 10^6^ cells/mL were stimulated with recombinant full-length proteins (10 μg/mL; in-house production N protein or M protein from AtaGenix Laboratories, Wuhan, China) or the RBDs (10 μg/mL) of different SARS-CoV-2 variants (8COV1 (Wuhan strain), HyTest, Russia; EVV00312 (Delta), and EVV00329 (BA.5), AntibodySystem, Schiltigheim, France). Stimulation was performed in the presence of anti-CD28 (0.5 μg/mL; Anti-Mouse CD28 Syrian hamster/IgG 37.51) and anti-CB49d (0.5 μg/mL; CD49d Monoclonal Antibody, Functional Grade, Rat/IgG2b, kappa (R1-2), (both from Invitrogen, Carlsbad, CA, USA)), and Brefeldin A (5 μg/mL; for 16 h. Cells stimulated with phorbol 12-myristate 13-acetate (Sigma-Aldrich, St Louis, MO, USA) and ionomycin (Thermo Fisher Scientific Inc., Waltham, MA, USA) were included as positive controls. After stimulation, cells were washed, then anti-CD16/32 was added for Fc-receptors blocking on ice for 10 min (purified anti-mouse CD16/32 AntibodyRat IgG2a, λ 93, Biolegend, San Diego, CA, USA). Then, cells were first stained with LIVE/DEAD aqua viability dye (Life Technologies GmbH, Darmstadt, Germany) and anti-mouse fluorochrome-conjugated antibodies for the surface markers (Biolegend, San Diego, CA, USA): anti-CD3-BV421 (Brilliant Violet 421™ anti-mouse CD3 Antibody, Rat IgG2b, κ, Clone 17A2), anti-CD8-BV650 (Brilliant Violet 650™ anti-mouse CD8a Antibody, Rat IgG2a, κ, 53-6.7), and anti-CD4-BV785 (Brilliant Violet 785™ anti-mouse CD4 Antibody, Rat IgG2a, κ, RM4-5) for 20 min in the dark at room temperature. After the wash step, cells were fixed and permeabilized using IntraPrep Permeabilization Reagent (Beckman Coulter, Fullerton, CA, USA). Subsequently, cells were washed again and stained with anti-mouse antibodies to intracellular markers anti-TNF-α-Alexa Flour 700 (Alexa Fluor^®^ 700 anti-mouse TNF-α Antibody, Rat IgG1, κ, MP6-XT22, Biolegend, San Diego, CA, USA), anti-IFN-γ-Alexa Flour 480 (Alexa Fluor™ 488, anti-mouse IFN gamma Antibody Rat/IgG1, kappa XMG1.2, Thermo Fisher, eBioscience Inc., San Diego, CA, USA), and anti-IL-2-PE (PE anti-mouse IL-2 Antibody, IgG2b κ, JES6-5H4, Thermo Fisher, eBioscience, Inc, San Diego, CA, USA) for 20 min in the dark at room temperature. Cells were then washed and were analyzed by a Cytoflex S flow cytometer (Beckman Coulter, Fullerton, CA, USA). Analysis of the flow cytometry data was performed using CytExpert software version 2.3.

### 2.7. RNA Extraction and RT-PCR Quantification of Viral RNA Copies and Plaque Assay

Lungs were harvested from mice 3 days post-infection. Following harvest, lungs were weighed, and then homogenized in sterile DMEM with gentamycin to generate a 20% lung-in-medium solution. Total RNA was extracted from lung homogenates using the ExtractRNA Reagent (Eurogen, Moscow, Russia) following the manufacturer’s instructions. Amplification and quantification of SARS-CoV-2 virus RNA were carried out by using a one-step quantitative reverse transcription polymerase chain reaction (RT-qPCR).

The infectious virus titer in the lungs was determined as previously described [26]. Briefly, 10-fold dilutions of lung homogenates were incubated for an hour on Vero E6 cells. Then, the supernatant was removed, and the cells were overlaid with 0.7% carboxymethylcellulose (Sigma-Aldrich, St. Louis, MO, USA) in DMEM. The plates were incubated at 37 °C for 72 h and virus plaques were counted. Results were expressed as Log10 plaque forming units (PFU) per mg lung tissue.

### 2.8. Virus-Neutralization Assay

Isolation, propagation, titration, and sequencing of the SARS-CoV-2 viruses used in this work were carried out as we previously described [27,28,29]. The virus titer was expressed as TCID_50_/mL (50% tissue culture infectious dose) and determined using the Reed and Muench method [30]. For virus neutralization analysis, sera from immunized animals were serially diluted in complete DMEM supplemented with 2% fetal bovine serum (FBS) and mixed with 1000 TCID_50_/mL of the corresponding SARS-CoV-2 variant and incubated at 37 °C for 1 h. After this, serum–virus mixtures were added to the monolayer of Vero E6 cells and incubated for 96 h. The cytopathic effect (CPE) was assessed visually. The neutralization titer was defined as the highest serum dilution inhibiting the development of CPE by at least 80%. The data obtained from the experiment were analyzed using GraphPad Prism 8.0 software.

### 2.9. Statistical Analysis

Statistical analysis was performed using the GraphPad Prism 8.0 software. Nonparametric tests were used throughout this paper for statistical analysis. Data were presented as the geometric mean with 95% confidence intervals or as median. Comparisons among groups were performed by using either the Mann–Whitney test (two groups) or Kruskal–Wallis test (multiple comparison) with Dunn’s post hoc test. Two-tailed *p* values were denoted, and *p* < 0.05 values were considered as significant.

## 3. Results

In this study, we investigated the immune responses caused by SARS-CoV-2 structural proteins (S, M, and N) encoded by mRNA-LNPs vaccine candidates, separately and in different combinations, to explore whether they influence each other and to what extent they mediate immunity. In addition, spike proteins were derived from evolutionarily distant SARS-CoV-2 variants (two from earlier ones: the wild-type (Wuhan-Hu-1) and Delta, and two from later ones: Omicron BA.5.3.1 and XBB.1) to study their abilities to induce an immune response.

Based on our recently studied mRNA platform [22], we have generated mRNA-LNP vaccines encoding the following full-length structural proteins derived from different SARS-CoV-2 variants:

- Spike (S) protein of the wild-type (Wuhan-Hu-1 strain);
- Spike (S) protein of the Delta variant (B.1.617.2);
- Spike (S) protein of the Omicron variant (BA.5.3.1);
- Spike (S) protein of the Omicron variant (XBB.1);
- N protein of the Omicron variant (BA.5.3.1);
- M protein of the Omicron variant (BA.5.3.1).

**Figure 1:**
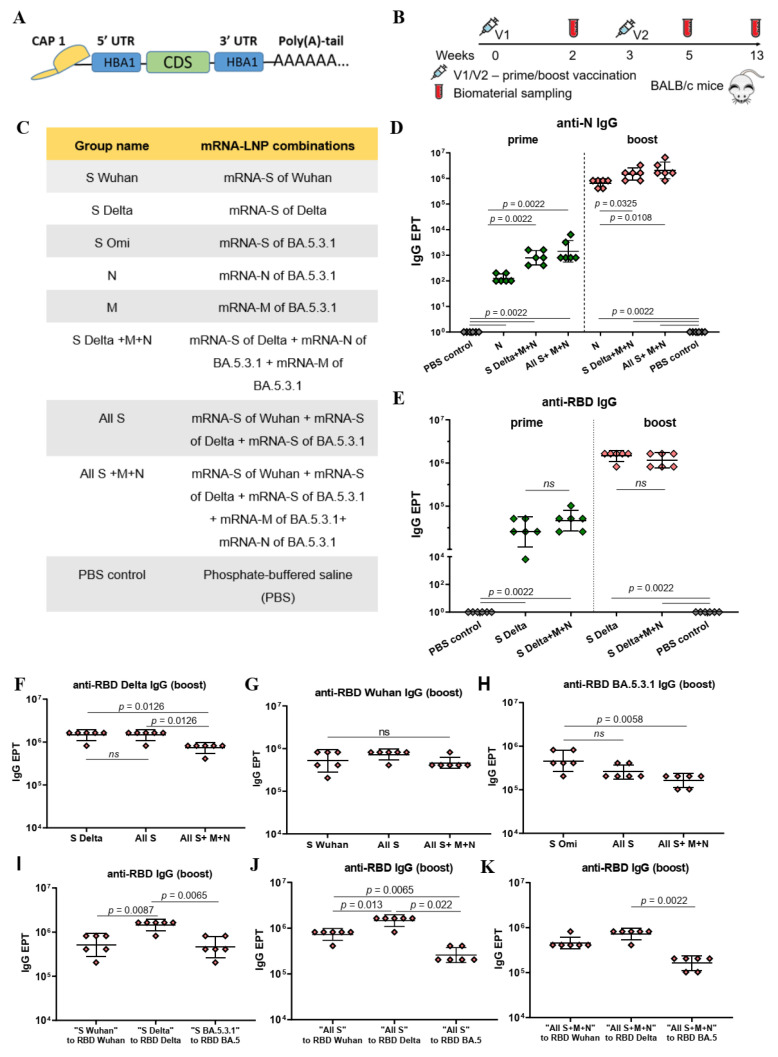
Structure of mRNA, immunogenicity experiment, mRNA-LNP vaccine compositions, and assessment of binding IgG antibodies. (**A**) Schematic representation of the mRNA platform used in this study. (**B**) Experimental design of immunogenicity assessment. Nine groups of BALB/c mice (*n* = 6 per group) were intramuscularly vaccinated with the mRNA-LNP combinations (5 μg of each mRNA per dose) or PBS (control group) at weeks 0 and 3. Two weeks after prime (V1) and booster (V2) vaccinations, serum samples were collected for analysis of binding antibody responses. Two and a half months after the booster mice were euthanized; serum and spleens were collected to determine virus neutralizing activity and T cell responses. (**C**) List of vaccine compositions and mouse group names. (**D**) Comparison of N-specific IgG endpoint titers (EPT) between PBS control group and vaccine groups after prime and booster vaccinations. (**E**) Comparison of RBD-specific IgG endpoint titers (EPT) between PBS control group and vaccine groups after prime and booster vaccinations. (**F**–**K**) Comparison of RBD-specific IgG endpoint titers (EPT) between vaccine groups after booster vaccination. EPT values are represented as scatter dot plots in logarithmic scale. Lines represent geometric mean with 95% confidence interval. Kruskal–Wallis test and the post hoc Dunn’s multiple comparisons test (**F**–**H**) or Mann–Whitney (**D**,**E**,**I**–**K**) test were used for statistical analysis (ns, not significant).

The coding regions for the investigated antigens were cloned into the linear bacterial plasmid pJAZZ-OK and supplemented with all the elements needed for the production of effectively translated mRNA molecules [22]. The transcribed mRNAs had the following structures which provided their stability and high translation rates: the cap-1 structure at the 5′ end; a 100-nt long poly(A)-tail at the 3′ end; the 5′ and 3′ UTRs from the human hemoglobin alpha subunit (HBA1) mRNA; and the coding sequences (CDS) of the protein of interest (Figure 1A). In addition, 100% uridines (U) were replaced with N1-methylpseudouridines (m1Ψ) to reduce mRNA immunogenicity. Thus, mRNAs encoding the full-length SARS-CoV-2 structural proteins were obtained (Figure S1).

The protein expression ability of all mRNA transcripts were examined in HEK293T cells. The cells were treated with the mRNAs and then lysed. An in-house bead-based immunoassay (Luminex^®^ xMAP^®^ technology) was used to detect the desired proteins. Corresponding monoclonal antibodies (specific to N and M proteins and the RBD of the S protein) were conjugated to the magnetic beads according to the Cookbook [23]. The assay was performed in the form of a “sandwich” non-competitive immunoassay. Median fluorescent intensity (MFI) was measured on a MAGPIX instrument and converted into ng/mL. The results confirmed the expression of investigating proteins in the cell lysate, as well as in the supernatant, demonstrating the functionality of our mRNA transcripts (Figure S2).

The next step was formulating mRNAs in lipid nanoparticles through the microfluidic mixing procedure. The lipid mixture consisted of an ionizable lipid (ALC-0315), DSPC, cholesterol, and a PEGylated lipid (ALC-0159) at molar ratios of 46.3:9:42.7:1.6, respectively. The formulated mRNA-LNPs were characterized by particle size, polydispersity, zeta potential, and mRNA encapsulation efficiency (Table S1). All mRNA-LNP sizes were below 100 nm (mean values ±SD were 76 ± 22.5, 75 ± 6, 69 ± 1.4 nm for mRNA-LNP coding S, N, M, respectively). The values of the polydispersity index did not exceed 0.15 and the zeta potential values were slightly negative, but not less than -10 mV. The average mRNA encapsulation efficiency was 86–91%.

The immunogenicity of mRNA-LNP was evaluated in BALB/c mice. Figure 1B represents the scheme of the experiment. Eight groups of mice (*n* = 6 per group) were vaccinated with various combinations of mRNA-LNPs (5 μg of each mRNA-LNP per dose), phosphate-buffered saline was injected into the mice in the control group (PBS control). The mRNA-LNPs combinations for immunization and the group names are listed in Figure 1C. Immunization was conducted intramuscularly at week 0 (V1) and week 3 (V2). Two weeks after prime vaccination (V1) and two weeks after the booster (V2), serum samples were collected for analysis of the binding antibody responses. Two and a half months after the booster mice were euthanized; the sera and spleens were collected to determine the virus-neutralizing activity and T cell responses (Figure 1B).

We determined binding IgG antibodies in the mouse serum samples after prime and boost immunizations through an in-house ELISA. Antibodies specific to N and M proteins and the RBD of the S protein were analyzed (Figure 1D–K). For that, ELISA plates were coated with recombinant N or M proteins (1 μg/mL) or RBDs from the SARS-CoV-2 variants under investigation (Wuhan, Delta, BA.5.3.1) in the same concentrations (0.5 μg/mL). Antibody endpoint titers (EPTs) were determined in serially diluted serum samples.

Compared to the PBS control group, strong N- or/and RBD-specific binding IgG responses (*p* < 0.005) were observed in all the groups immunized with mRNA-LNP coding N or/and S proteins, respectively, both after the prime and boost vaccinations (Figure 1D,E). The geometric mean titers (GMTs) of the N- and RBD-specific IgG after booster dose were three orders of magnitude higher than after prime immunization in all mRNA-LNP vaccinated groups (*p* = 0.0022; *p* = 0.0048 (Figure S3)). The maximum values were: 1 459 648 for anti-RBD Delta IgG in the groups “All S” and “S Delta” and 2 064 255 for anti-N IgG in the “All S+M+N” group.

The GMT for N- specific binding IgG in the “N” group was less than those for the groups “S Delta+M+N” and “All S+M+N” after both the prime (*p* = 0.0022) and boost (*p* < 0.05) doses (Figure 1D). The data suggest that the addition of mRNAs encoding S and M antigens to the mRNA-N in the vaccine composition enhances the immunogenicity of mRNA-N, inducing a more robust humoral response to the N protein. This can be observed after both prime and boost vaccinations (Figure 1D). Conversely, no significant difference was found when comparing GMTs for RBD-specific binding IgG in the “S Delta” and “S-Delta+M+N” groups. (Figure 1E). Thus, the addition of mRNAs encoding the N and M proteins in the mRNA-S vaccine preparation did not affect the humoral response caused by mRNA-S alone (Figure 1E). Similarly, comparing GMTs for RBD-specific binding IgG in the groups “S Delta” and “All S” did not reveal significant differences (Figure 1F). Furthermore, combining the three mRNAs encoding the S proteins of all variants used (Wuhan, Delta, Omicron BA.5.3.1) did not lead to an increase in the GMT of RBD-binding antibodies in the “All S” group compared to those induced by mRNA-S alone (Figure 1F–H). However, the vaccine composition comprising all used mRNAs (encoding three S protein, N and M) contributed to a lower GMT of RBD-binding IgG obtained for the “All S+M+N” group compared to those induced by the mRNA-S of Delta (*p* = 0.0126) or the mRNA-S of Omicron alone (*p* = 0.0058), but not that induced by the mRNA-S of Wuhan (Figure 1F–H).

Furthermore, it was found that the GMT of the RBD-binding IgG induced in the “S Delta” group was higher than those produced in the “S Wuhan” (*p* = 0.0087) or “S Omi” groups (*p* = 0.0065) (Figure 1I). In the “All S” group, the GMT of the binding IgG that was specific to the RBD of Delta was higher than the GMT of the binding IgG that was specific to the RBD of Wuhan (*p* = 0.013) or the RBD of Omicron BA.5.3.1 (*p* = 0.022) (Figure 1J). Similarly, in the “All S+M+N” group the GMT of the binding IgG that was specific to the RBD of Delta was higher than the GMT of the binding IgG that was specific to the RBD of Omicron BA.5.3.1 (*p* = 0.0022) (Figure 1K).

As for the M-specific antibodies, poor responses were observed in all mRNA-M-vaccinated groups after both prime and booster vaccination by bead-based immunological analysis and no responses were observed by ELISA analysis. M-specific binding antibodies were only detected in the “M” group after the booster compared to the PBS control group (Figure S4).

**Figure 2:**
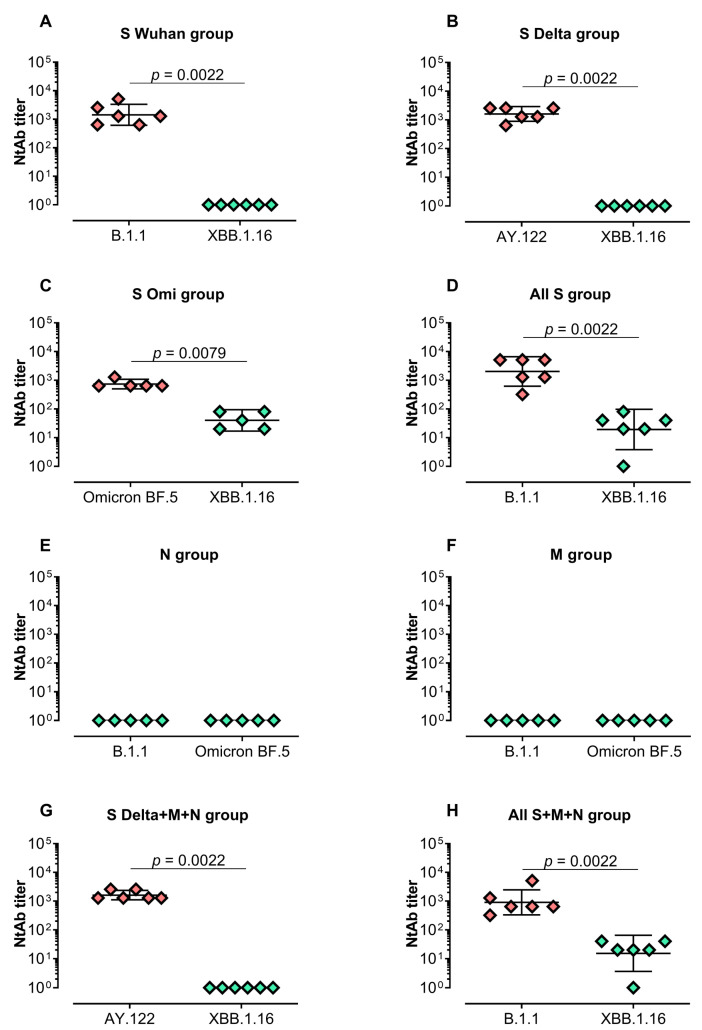
Serum-neutralizing activity against B.1.1, Delta, Omicron BF.5, or XBB.1.16 in vaccinated BALB/c mice. Neutralizing antibody (NtAb) titers against B.1.1 and XBB.1.16 in the groups “S Wuhan”, “All S” and “All S+M+N” (**A**,**D**,**H**); NtAb titers against AY.122 and XBB.1.16 in the groups “S Delta” and “S Delta+M+N” (**B**,**G**); NtAb titers against Omicron BF.5 and XBB.1.16 in “S Omi” group (**C**); NtAb titers against B.1.1 and Omicron BF.5 in the groups “M” and “N” (**E**,**F**). Neutralizing antibody titers were determined by the highest plasma dilution protecting 80% of the infected wells. Titer values are represented as scatter dot plots in logarithmic scale. Lines represent geometric means with 95% confidence interval. Mann–Whitney test was used for statistical analysis.

Next, we evaluated the virus-neutralizing activity in serum samples (Figure 2). A high level of neutralizing activity was detected in the groups vaccinated with mRNA-S alone (“S Wuhan”, “S Delta” and “S Omi” groups) against closely related strains (Wuhan-like strain B.1.1, Delta sublineage AY.122 or Omicron BF.5, respectively) (Figure 2A–D). The GMTs of the neutralizing antibodies in these groups were 1 437, 1 613, and 735, respectively. However, the neutralizing activity was not detectable (Figure 2A,B) or significantly lower (Figure 2C, *p* = 0.0079) against the evolutionarily distant variant Omicron XBB.1.16. Similar results were demonstrated in the groups “All S” and “All S+M+N”—high GMT values against B.1.1 and reduced activity for XBB.1.16 (Figure 2D,G,H, *p* = 0.0022).

As expected, no neutralizing activity was detected in mice groups vaccinated with mRNA-N or mRNA-M alone without the addition of mRNA-S in the vaccine composition (Figure 2E,F).

**Figure 3:**
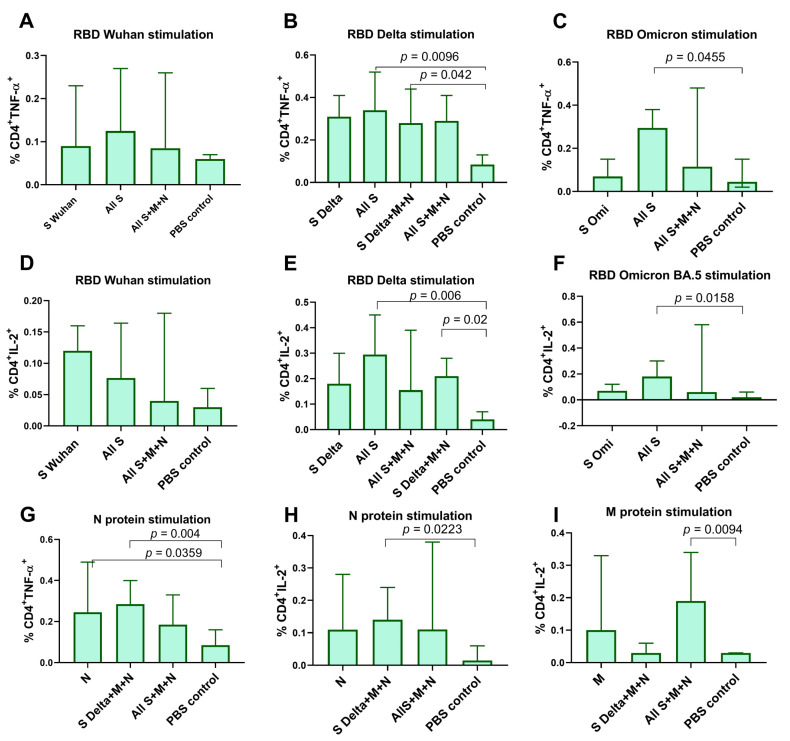
Vaccine-specific T cell responses in mouse spleens measured by intracellular cytokine staining (ICS). Splenocytes were stimulated with full-length N or M protein or the RBD of S protein, followed by ICS and flow cytometry analysis. (**A**–**F**) Comparison of percentage of cytokine-positive, RBD-specific CD4^+^ T cells between vaccinated groups and PBS control (percentage of total CD4^+^ T cells). (**G**–**I**) Comparison of percentage of cytokine-positive, N-, M-specific CD4^+^ T cells between vaccinated groups and PBS control. Data are presented as median with 95% confidence interval. Kruskal–Wallis test and the post hoc Dunn’s multiple comparisons test was used for statistical analysis.

M-, N-, and S-specific T cell responses were measured by intracellular cytokine staining (ICS) after the stimulation of splenocytes with full-length M or N proteins or the RBD of the spike protein, respectively. Specific CD4^+^ T cell lymphocyte producing IL-2 and TNF-α were predominantly detected and were significantly higher compared to the PBS control group (Figure 3). As for IFN-γ and CD8^+^ T cell activation, we did not find any significant differences between the studied groups. Among the mRNA-S-vaccinated groups RBD-specific responses were observed in the “All S” (*p* < 0.01) and “S Delta+M+N” (*p* < 0.05) groups but not in the “All S+M+N” group, mostly when stimulated by the RBD of the Delta variant (Figure 3A–F). Similarly, N-specific CD4^+^ T cells predominantly expressed TNF-α and IL-2 compared to the PBS control group (*p* < 0.05) (Figure 3G,H). Among all of the mRNA-N-vaccinated groups, N-specific CD4^+^ T cell responses were more pronounced in the “S Delta+M+N” group relative to the PBS control (*p* = 0.004 for TNF-α^+^CD4^+^ T cells, *p* = 0.0223 for IL-2^+^CD4^+^ T cells) (Figure 3G,H). For the M protein, IL-2-producing CD4^+^ T cells were only detected in the “S Delta+M+N” group compared to the PBS control group, *p* = 0.0094 (Figure 3I).

The data obtained from the immunogenicity experiment have shown the potential of including mRNA-N rather than mRNA-M in the mRNA-LNP vaccine. Furthermore, compared to mRNA-N alone, the combination of mRNAs encoding N and S proteins in the vaccine composition allows for the induction of a more robust immune response to N protein from both humoral and cell-mediated immunity.

**Figure 4:**
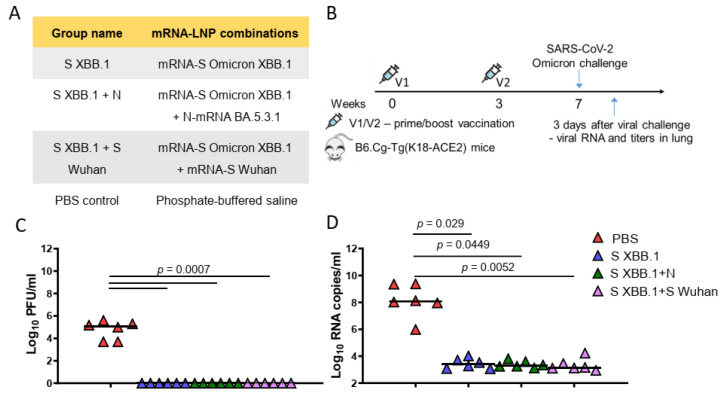
Mice SARS-CoV-2 challenge experiment. (**A**) List of vaccine candidates’ compositions and group names. (**B**) Experimental design of challenge experiment. Four groups of B6.Cg-Tg(K18-ACE2) mice (*n* = 15 per group) were intramuscularly immunized with mRNA-LNP combination (5 μg of each mRNA per dose) or PBS (control group) at weeks 0 and 3 and were intranasally challenged with aSARS-CoV-2 BF.7 strain (10^5^ TCID_50_) at week 7. On day 3 after challenge (*n* = 6), lung tissues were harvested for analysis of viral RNA copies, viral titers. (**C**) Comparison of viral titers (plaque-forming unit (PFU)/mL) in the mouse lungs between different groups. (**D**) Comparison of viral RNA copies in the mouse lungs (log10 RNA copies per/mL) between different mice groups. Data are represented as scatter dot plots in logarithmic scale. Lines represent medians, Kruskal–Wallis test and the post hoc Dunn’s multiple comparisons test was used.

Then, we evaluated the protective efficacy of mRNA-LNP vaccines encoding S and N proteins compared to commonly used and approved types of mRNA-LNP vaccines—encoding S alone or two variants of the S protein (bivalent mRNA-LNP vaccine). For this comparison, we used an mRNA encoding the S protein from the recently circulating Omicron XBB.1. For the bivalent mRNA-LNP vaccine, an mRNA encoding the S protein of the Wuhan strain was added to mRNA-S XBB.1. The mRNA-LNP combinations used for immunization and the group names are listed in Figure 4A. Vaccine efficacy was studied on transgenic mice, of lineage B6.Cg-Tg(K18-ACE2), expressing the ACE2 receptor for virus entry. The experimental design is represented in Figure 4B. Three groups of mice were immunized at week 0 (prime) and week 3 (boost) with mRNA-LNPs (5 μg of each mRNA-LNP per dose), while the control group of mice was injected with PBS (PBS control group). The mice were then challenged intranasally with the Omicron BF.7 strain (10^5^ TCID_50_) of SARS-CoV-2 at week 7. Viral titers and RNA copies in the lungs were measured 3 days after the challenge (Figure 4B). Compared to the PBS control group, all mRNA-LNP compositions (mRNA-S alone, combined with mRNA-N or bivalent vaccine) induced total viral control with no detectable infectious virus being found in the lungs (*p* = 0.0007) (Figure 4C). In addition, all mRNA-LNP compositions demonstrated a high efficacy in reducing the number of copies of viral RNA in the lungs compared to the control group (*p* < 0.05) (Figure 4D). However, neither the viral titers measurement nor the quantification of viral RNA by the more-sensitive reverse transcription polymerase chain reaction (RT-qPCR) method revealed any differences between the mRNA-LNP vaccinated groups.

## 4. Discussion

Recently, we studied an mRNA-LNP platform which provided an efficient long-term expression of the encoded gene in vivo through both intramuscular and intravenous administration. In this study, we have demonstrated the potential of our mRNA-LNP platform for the development of COVID-19 vaccine candidates.

Based on our platform, we have generated mRNA-LNP formulations encoding the structural proteins of SARS-CoV-2. We conducted comprehensive investigations into the immunogenicity and protective efficacy of mRNA-LNPs encoding M, N, or S proteins, both individually and in various combinations. We also examined how they mutually influence each other to elicit specific immune responses.

It is known that mRNA vaccines against COVID-19 are capable of inducing robust CD4^+^ and CD8^+^ T cell responses and strong antibody responses [31,32]. The data obtained in our study demonstrated the strong potential of using our mRNA-LNP platform in vaccine development. All of the studied vaccine compositions which encoded S and N proteins induced high GMT of RBD- and N-specific IgG antibodies in mice (the GMT for anti-RBD IgG was averaged 10^6^). In addition, T cell responses that were mainly in the form of specific CD4^+^ T cell lymphocytes producing IL-2 and TNF-α were detected. As for IFN-γ and CD8^+^ T cell activation, we did not find any significant differences between the studied groups. In general, the T cell responses we obtained were poorly pronounced and we suppose that this was due to the splenocytes being stimulated with a full-length N protein and RBD rather than peptide pools.

Contrary to expectations, a poor immune response was observed when the mice were vaccinated with an mRNA-LNP encoding the M protein. However, the limited data confirm that its immunogenic properties caused humoral [33] and strong T cell [16,34] responses.

We also obtained the expected results of virus-neutralizing activity in vaccinated mice. A significant level of neutralizing activity was detected in the groups vaccinated with mRNA-S alone or the combination of three mRNA-S against closely related strains but was not detectable or significantly lower against the evolutionarily distant variant Omicron XBB.1.16. The data reflect the problem of the ongoing evolution of SARS-CoV-2 in the human population which has resulted in the emergence of variants of concern (VOCs). It has been found that, before the emergence of Omicron, VOCs such as Alpha and Delta had increased transmissibility and had modest degrees of immune evasion. However, the large number of additional mutations in the Omicron spike gene gave it an advantage in immune evasion and led to the displacement of Delta by Omicron. Further evolution of the Omicron lineage (BA.2, BA.4, BA.5, XBB) has resulted in significant reductions in antibody neutralization titers in sera from naturally infected or vaccinated individuals. [35].

Today, due to the ongoing emergence of new SARS-CoV-2 variants, the global vaccine strategy aimed at updating the S protein in vaccines (the introduction of bivalent or monovalent boosters) still remains the most relevant. However, this approach is too slow to be ideal. Therefore, the development of next-generation vaccine strategies for broader and long-lasting protection is equally important.

For this purpose, several studies have tested SARS-CoV-2 candidate vaccines that include other structural proteins (mostly the N protein) in the adenoviral vectors [20,36], modified vaccinia Ankara vectors [37,38], subunit protein vaccine candidates [39,40], and others [41]. These studies have demonstrated the protective efficacy of and the T-cell and humoral responses induced by vaccination. However, there has been no comparison of their efficacy with the clinically proven S-targeted vaccines, e.g., mRNA vaccines. In addition, the efficacy of most of them against the new VOCs remains unclear.

As for mRNA vaccines, in one study the authors created an mRNA-LNP encoding the full-length N and S proteins of SARS-CoV-2 (Wuhan-Hu-1 strain). They showed that, compared to mRNA-S alone, vaccination with a combination of mRNA-S+N induced a more robust control of the Delta and Omicron variants in the lungs and also provided enhanced protection to the upper respiratory tract [42].

In our study, we obtained some interesting results on humoral and cellular responses. Although the amount of each mRNA component was identical in all of the mRNA-LNP vaccines used (5 μg), some differences in the strength of the immune responses to the expressed antigens were seen between groups. Our data demonstrated that the addition of mRNAs encoding the S and M antigens to mRNA-N in the mRNA-LNP vaccine enhanced immunogenicity of mRNA-N, inducing a more robust humoral response to the N protein. A similar pattern was observed in T cell responses following the stimulation of splenocytes with the full-length N protein. Among the all groups vaccinated with mRNA-N, N-specific CD4^+^ T-cell responses were most pronounced in the “S Delta+M+N” group compared to the group vaccinated with mRNA-N alone. Based on our results, we suggest that, in our mRNA-LNP vaccine compositions, the mRNA that encoded S protein played a key role in enhancing the anti-N protein immune response. Conversely, the addition of mRNAs encoding the N and M proteins to the mRNA-S in the mRNA-LNP vaccine did not affect the humoral response caused by mRNA-S. Furthermore, the combination of three mRNAs encoding the S protein of all variants used (Wuhan, Delta, Omicron BA.5.3.1) did not increase the GMT of RBD-binding antibodies in the “All S” group compared to those induced by mRNA-S alone.

It was also noticed that the GMTs of anti-RBD Delta IgG induced in the “S Delta”, “All S” and “All S+M+N” groups were higher than the GMTs of the IgG specific to the RBDs of the Wuhan or Omicron variants in the same groups. Numerous studies have indicated the higher intrinsic disease severity caused by the Delta variant compared with the Omicron variant [43,44,45,46], although both have the same high transmissibility [47]. So, we suppose that the high virulence of the Delta variant may contribute to a more pronounced immune response compared to the others as we found in our study.

The same phenomena, but with the opposite effect, were previously observed in the study by Hajnik et al. [42]. They also showed that mRNA-N alone in the mRNA-LNP vaccine was highly immunogenic and induced robust N-specific T cell responses and binding antibodies. In contrast to our results, vaccination with the combination of mRNA-S+N resulted in enhanced S-specific immunity in the T-cell response compared to mRNA-S alone. However, the authors did not compare mRNA-N and mRNA-S+N in mRNA-LNP compositions using the same experiment as we did. The authors suggested that cross-priming effects may occur between N and S antigens after vaccination, or that mRNA-N co-immunization may induce an immune environment that enhances S-specific immunity (or, conversely, as we found in our study).

Another study [48] demonstrated the additional enhancement of T-cell responses to SARS-CoV-2 structural M and N proteins after S-mRNA vaccination in individuals with previous SARS-CoV-2 infection and in infection-naive individuals with cross-reactivity to the seasonal coronaviruses. It was suggested that the cellular response was not exclusively directed towards the S-related epitopes after spike mRNA vaccination. It remains controversial how antigen recognition by T cells can be so exceptionally specific [49]. The data suggest that individual T-cell clones can have cross-reactivity and recognize different epitopes [50].

In the challenge experiment we compared mRNA-LNP vaccines encoding S and N proteins with commonly used and approved types of mRNA-LNP vaccines—coding S alone or a bivalent mRNA-LNP vaccine. All of the mRNA-LNP compositions have shown excellent results in terms of protective efficacy: total viral control with no detectable infectious virus in the lungs and reducing copies of viral RNA in the lungs compared to the PBS control group three days after intranasal challenge with the Omicron subvariant BF.7. High transmissibility and a significant resistance to neutralizing antibodies induced by COVID-19 vaccines and boosters characterize this subvariant [51]. The mRNA-LNP vaccine encoding the S protein of XBB.1 alone has sufficient protective properties without the addition of mRNAs encoding the other proteins (S or N) to the vaccine composition. However, we did not investigate the protective efficacy of these same vaccine compositions when the mice were challenged with an evolutionarily distant variant (for example, Delta, or highly mutated BA.2.86). This is our main limitation. Performing a similar experiment in the future might help to reveal the differences between the compositions and the possible benefits of adding the more conservative N protein in our mRNA-LNP vaccine.

## 5. Conclusions

Our study serves as a compelling demonstration of the potential utility of our mRNA-LNP platform in vaccine development, using the SARS-CoV-2 infection as an illustrative example. The mRNA-LNP vaccine compositions encoding the structural proteins (S and N) have demonstrated a robust immunogenicity in mice, eliciting both humoral and cellular immune responses. Additionally, we have elucidated that the presence of different protein-encoding mRNAs within the mRNA-LNP vaccine compositions could exert mutual influence, resulting in variable degrees of immune response strength compared to mRNA encoding individual proteins. Based on our results, we suggest that, in our mRNA-LNP vaccine compositions, the S protein plays a key role in enhancing the immune response to the N protein.

In addition, our mRNA-LNP vaccine compositions encoding the S protein demonstrated a high level of virus neutralizing-activity against homologous variants, but it was not detectable or significantly lower against the evolutionarily distant, recently dominant XBB.1.16. These data once again confirmed the need to update the S protein-based vaccine.

Updating the S protein-based vaccines to account for circulating variants of SARS-CoV-2 remains the main strategy for obtaining effective vaccine formulations until a new strategy is developed that can provide a broad protection against new emerging variants. For this purpose, a deeper understanding of the development of adaptive immunity to the SARS-CoV-2 infection is essential. Our study has shown that more detailed studies are needed on the mechanisms of S-, N-, M-specific immunity formation after combined mRNA vaccination, such as the protein expression, antigen presentation, and the stimulation of the innate response, the formation of the T- and B-cell pool, their clonal diversity, and the specificity of their interactions with antigens.

## Data Availability

The data presented in this study are available on request from the corresponding author.

## Supplementary Materials

The following supporting information can be downloaded at: https://www.mdpi.com/article/10.3390/vaccines12040379/s1, Figure S1: Schematic representation of mRNAs, encoding SARS-CoV-2 structural proteins using in this study; Figure S2: mRNAs translation check on HEK293T cells; Table S1: Physicochemical properties of mRNA-LNP formulations; Figure S3: ELISA results for binding IgG after prime (V1) and boost (V2) vaccinations; Figure S4: Bead-based immunoassay results for M-specific binding IgG after prime (V1) and boost (V2) vaccinations.
