# Supplementary material for: Major Role of S-Glycoprotein in Providing Immunogenicity and Protective Immunity in mRNA Lipid Nanoparticle Vaccines Based on SARS-CoV-2 Structural Proteins
Source: Vaccines (Basel). 2024 Apr 2;12(4):379. doi: 10.3390/vaccines12040379 (PMC11053793; doi:10.3390/vaccines12040379)
Supplement: Supplementary file 1 [file vaccines-12-00379-s001.zip › vaccines-2887678-supplementary.pdf]

SUPPLEMENTARY

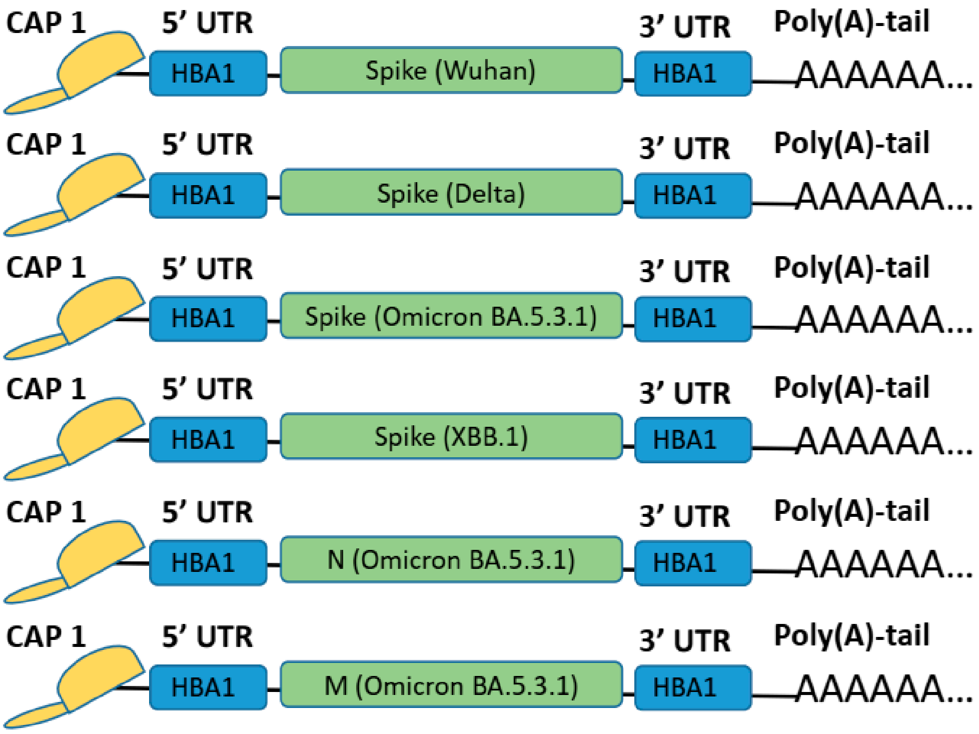

Figure S1. Schematic representation of mRNAs, encoding SARS-CoV-2 structural proteins using in this study.

MILLIPLEX Analyst Detail Report

4-P.log.weighted(3.89, 10.26, 0.71, 0.22)
Chi=8.31%, CV=0.45%, R²=0.999, DC=(0.13, 910.10)

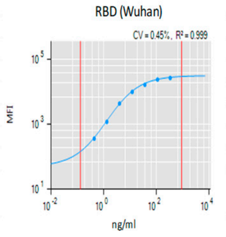

Samples	MFI	ng/ml
Blank	25	<0.13↓
S1 (300 ng/ml)	25185	287,23
S2 (100 ng/ml)	22440	113,62
S3 (33,33 ng/ml)	15545	28,76
S4 (11,11 ng/ml)	9374	10,87
S5 (3,7 ng/ml)	4205	4,04
S6 (1,23 ng/ml)	1155	1,22
S7 (0,41 ng/ml)	356,5	0,41
RBD (Wuhan) in supernatant	10110	**12.23**
RBD (Wuhan) in lysate	16526	**33.84**

5-P.log(2.02, 10.46, 0.30, -0.01, 0.70)
DC=(0.19, 577.52) Chi = 3.32%

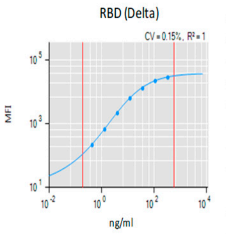

Samples	MFI	ng/ml
Blank	23	<0.19↓
S1 (300 ng/ml)	26615	295,64
S2 (100 ng/ml)	20786	105,38
S3 (33,33 ng/ml)	12225	31,3
S4 (11,11 ng/ml)	6016	11,37
S5 (3,7 ng/ml)	2072	3,67
S6 (1,23 ng/ml)	655	1,25
S7 (0,41 ng/ml)	212	0,41
RBD (Delta) in supernatant	12572	**32,89**
RBD (Delta) in lysate	18662	**77,06**

5-P.linear.weighted(22.44, 19536.86, 3.60, 30.54, 0.70)
DC=(0.64, 433.26) Chi = 5.35%

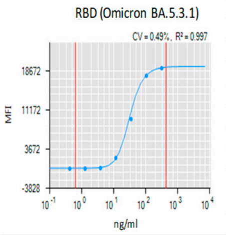

Samples	MFI	ng/ml
Blank	21	<0.64↓
S1 (300 ng/ml)	19284	299,99
S2 (100 ng/ml)	17842	101,49
S3 (33,33 ng/ml)	9540	29,85
S4 (11,11 ng/ml)	2076	12,11
S5 (3,7 ng/ml)	145	3,72
S6 (1,23 ng/ml)	30	1,2
S7 (0,41 ng/ml)	23	<0.64↓
RBD (BA.5.3.1) in supernatant	1206	**9,49**
RBD (BA.5.3.1) in lysate	5124	**19,22**

MILLIPLEX Analyst Detail Report

5-P.log.weighted(3.09, 10.43, 0.27, 4.72, 1.99)
DC=(0.28, 501.55) Chi = 32.26%

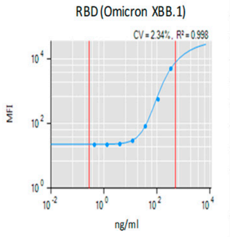

Samples	MFI	ng/ml
Blank	23,8	<11.11↓
S1 (300 ng/ml)	4652	310,39
S2 (100 ng/ml)	528	101,49
S3 (33,33 ng/ml)	81	29,85
S4 (11,11 ng/ml)	29	12,11
S5 (3,7 ng/ml)	23	3,72
S6 (1,23 ng/ml)	22	1,2
S7 (0,41 ng/ml)	22	<0.64↓
RBD (XBB.1) in supernatant	14	**<0.28↓**
RBD (XBB.1) in lysate	233,25	**61,09**

5-P.linear.weighted(0.00, 36722.73, 2.17, 120.24, 0.89)
DC=(0.27, 353.34) Chi = 14.47%

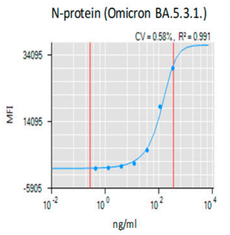

Samples	MFI	ng/ml
Blank	51,5	<0,27↓
S1 (300 ng/ml)	29982	289,9
S2 (100 ng/ml)	18541	121,79
S3 (33,33 ng/ml)	5565	31,66
S4 (11,11 ng/ml)	1598	8,07
S5 (3,7 ng/ml)	748	3,46
S6 (1,23 ng/ml)	291	1,2
S7 (0,41 ng/ml)	123	0,46
N-protein (BA.5.3.1) in supernatant	2096	**10,9**
N-protein (BA.5.3.1.) in lysate	19058	**126,33**

5-P.linear.weighted(34.68, 14656.44, 1.21, 305.02, 0.01)
DC=(1.23, 328.98) Chi = 38.86%

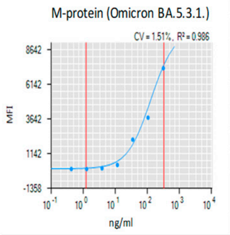

Samples	MFI	ng/ml
Blank	75,5	<1,3↓
S1 (300 ng/ml)	7222	289
S2 (100 ng/ml)	3704	81
S3 (33,33 ng/ml)	2144	42,1
S4 (11,11 ng/ml)	338	7
S5 (3,7 ng/ml)	100	1,92
S6 (1,23 ng/ml)	49	0,54
S7 (0,41 ng/ml)	44	0,38
M-protein (BA.5.3.1) in supernatant	89,5	**1,66**
M-protein (BA.5.3.1.) in lysate	147	**3,03**

Figure S2. mRNAs translation check on HEK293T cells. Bead based immunoassay results. Median fluorescent intensity (MFI) was measured on a MAGPIX instrument and converted in ng/ml. For each identified analyte (RBD, N or M), the MFI-value was converted to ng/mL by interpolation from a 5-parameter logistic (5-PL) curve of reference standard using the MILLIPLEX® Analyst 5.1 software (The Life Science/Merck KGaA).

Table S1. Physicochemical properties of mRNA-LNP formulations.

	mRNA-S-LNP				mRNA-N-LNP				mRNA-M-LNP			
	d (nm)	PDI	Z (mV)	E%	d (nm)	PDI	Z (mV)	E%	d (nm)	PDI	Z (mV)	E%
	76	0,128	-8	92								
	76	0,093	-6	83								
	73	0,108	-8	84								
	78	0,146	-8	92								
	74	0,121	-8	92								
	80	0,112	-7	89								
	73	0,111	-8	88	70	0,104	-7	91	68	0,14	-8	92
	78	0,102	-9	84	79	0,148	-8	82	70	0,13	-5	90
Mean	76	0,115	-8	88	75	0,126	-8	86	69	0,135	-7	91
SD	2,563	0,016	0,886	3,891	6,364	0,031	0,707	4,583	1,414	0,007	2,121	1,414
Median	76	0,112	-8	89	75	0,126	-8	85	69	0,135	-7	91

The diameter (d) and polydispersity index (PDI) and Zeta potential (Z) of the mRNA-LNP formulations were measured by dynamic light scattering (for d and PDI values) or by determining the electrophoretic mobility (for Z) using a Zetasizer Nano ZS instrument (Malvern Panalytical). E% – encapsulation efficiency of mRNA in lipid nanoparticles.

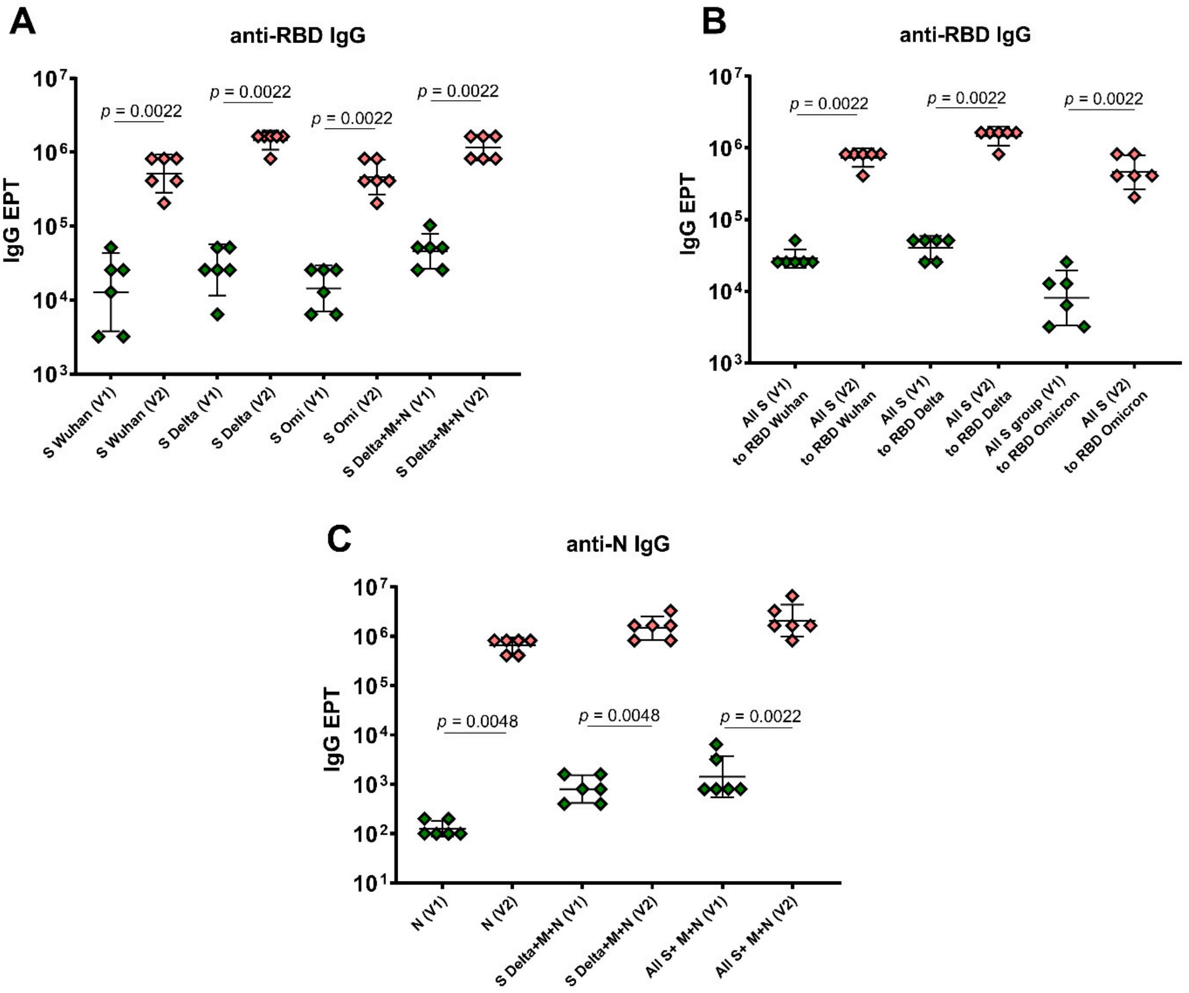

Figure S3. ELISA results for binding IgG after prime (V1) and boost (V2) vaccinations. Serum RBD-specific (**A-B**) and N- (**C**) binding IgG after prime (V1) and boost (V2) vaccinations. EPT values are represented as scatter dot plots in logarithmic scale. Lines represent geometric means with 95% confidence interval. Mann-Whitney test was used for statistical analysis.

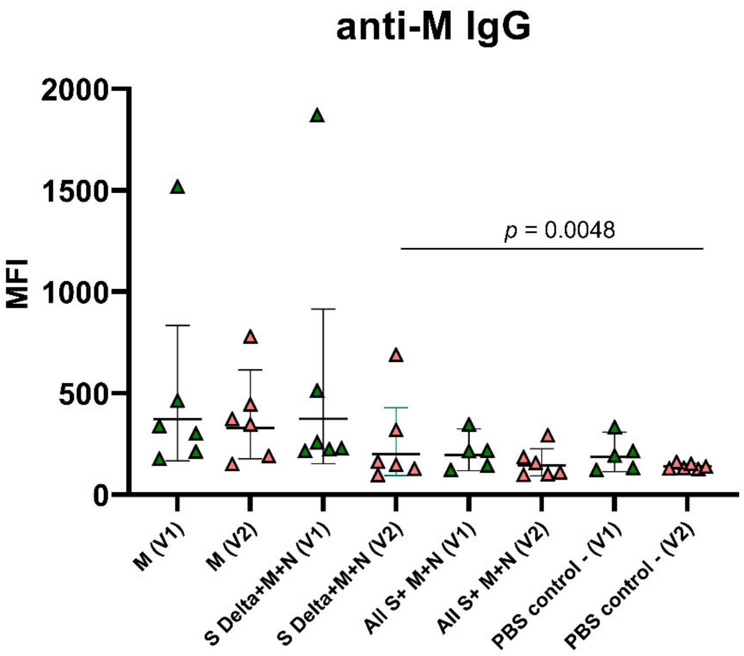

Figure S4. Bead-based immunoassay results for M-specific binding IgG after prime (V1) and boost (V2) vaccinations. MFI – median fluorescent intensity. Mann-Whitney test was used for statistical analysis.
